# Effects of Gangliosides on Spermatozoa, Oocytes, and Preimplantation Embryos

**DOI:** 10.3390/ijms21010106

**Published:** 2019-12-22

**Authors:** Bo Hyun Kim, Won Seok Ju, Ji-Su Kim, Sun-Uk Kim, Soon Ju Park, Sean M. Ward, Ju Hyeong Lyu, Young-Kug Choo

**Affiliations:** 1 CHA Fertility Center, 5455 Wilshire Blvd. Los Angeles, CA 90036, USA; kimbh12@hanmail.net; 2 Department of Biological Science, College of Natural Sciences, Wonkwang University, 460, Iksan-daero, Iksan-si, Jeollabuk-do 54538, Korea; jws7895@naver.com (W.S.J.); sjpark75@wku.ac.kr (S.J.P.); 3 Primate Resources Center (PRC), Korea Research Institute of Bioscience and Biotechnology, Neongme-gil, Ibam-myeon, Jeongup-si, Jeonvuk 56216, Korea; kimjs@kribb.re.kr; 4 National Primate Research Center, Korea Research Institute of Bioscience and Biotechnology, 30, Yeonggudanji-ro, Ochang-eup, Cheongwon-gu, Cheongju-si, Chungcheongbuk-do 28116, Korea; sunuk@kribb.re.kr; 5 Department of Physiology and Cell Biology, University of Nevada, Reno School of Medicine, Reno, NV 89557, USA; smward@med.unr.edu (S.M.W.); jlyu@med.unr.edu (J.H.L.); 6 Institute for Glycoscience, Wonkwang University, 460, Iksan-daero, Iksan-si, Jeollabuk-do 54538, Korea

**Keywords:** ganglioside, spermatozoa, oocyte, embryo, maturation, apoptosis

## Abstract

Gangliosides are sialic acid-containing glycosphingolipids, which are the most abundant family of glycolipids in eukaryotes. Gangliosides have been suggested to be important lipid molecules required for the control of cellular procedures, such as cell differentiation, proliferation, and signaling. GD1a is expressed in interstitial cells during ovarian maturation in mice and exogenous GD1a is important to oocyte maturation, monospermic fertilization, and embryonic development. In this context, GM1 is known to influence signaling pathways in cells and is important in sperm–oocyte interactions and sperm maturation processes, such as capacitation. GM3 is expressed in the vertebrate oocyte cytoplasm, and exogenously added GM3 induces apoptosis and DNA injury during in vitro oocyte maturation and embryogenesis. As a consequence of this, ganglioside GT1b and GM1 decrease DNA fragmentation and act as H_2_O_2_ inhibitors on germ cells and preimplantation embryos. This review describes the functional roles of gangliosides in spermatozoa, oocytes, and early embryonic development.

## 1. Introduction

According to membrane theory, the plasma membrane is a mosaic bilayer in which dynamic changes in biological activity are mediated by proteins, lipid rafts, cholesterol, and sphingolipids [1,2,3]. Lipid rafts are sphingolipid and cholesterol-enriched plasma membrane microdomains that serve as platforms for protein segregation in the membrane [4]. Effective changes in the functional biology and molecular organization of the plasma membrane are induced by lipid raft aggregation, disaggregation, and differential partitioning kinetics between lipid-ordered and lipid-disordered regions of the plasma membrane [5]. These changes are important to establish polarity and asymmetry in membrane activities [6,7].

Gangliosides are sialic acid-containing acidic glycosphingolipids. Gangliosides are found in animal plasma membranes and are especially abundant in nervous cell membranes [8]. Many types of gangliosides have been separated from the brain in different species, and GM1, GD1a, GD1b, and GT1b comprise the large majority of gangliosides in the brains of all mammal species investigated [9]. Gangliosides are implicated in the control of cellular functions, serving as antigens, and play roles in cellular adhesion and signal transduction [10]. Several gangliosides have been demonstrated to regulate cell differentiation, cell growth, and play significant roles in the immune defense system. Overexpression of gangliosides in patients with renal cell carcinomas has been related to multiple T cell dysfunctions [11]. Elevated levels of GD3 found in ovarian cancer ascites fluid inhibit NKT cell activation [12] and apoptosis of T cells [13].

Reactive oxygen species (ROS) generation has been detected in reproductive and non-reproductive cells and modulated cellular survival and death. Hydrogen peroxide (H_2_O_2_) increases apoptosis by interrupting the antioxidant defense system during oocyte maturation and embryonic development in vitro [14,15]. The ability of various gangliosides to diminish lipid peroxidation accumulation and free radical scavenging has been explained in isolated rat myocardiocytes [16] and brain cells [17,18,19,20,21]. Exogenous application of gangliosides has been shown to affect biological events and cell properties, including protection against the oxidative stress of the cell. For example, treatment with GM1 protects against oxidative stress-induced neuronal cell injury and traumatic injury-induced brain edema [22]. Supplementation of GT1b to culture medium also protects human spermatozoa from DNA damage induced by ROS [23]. 

**Figure 1:**
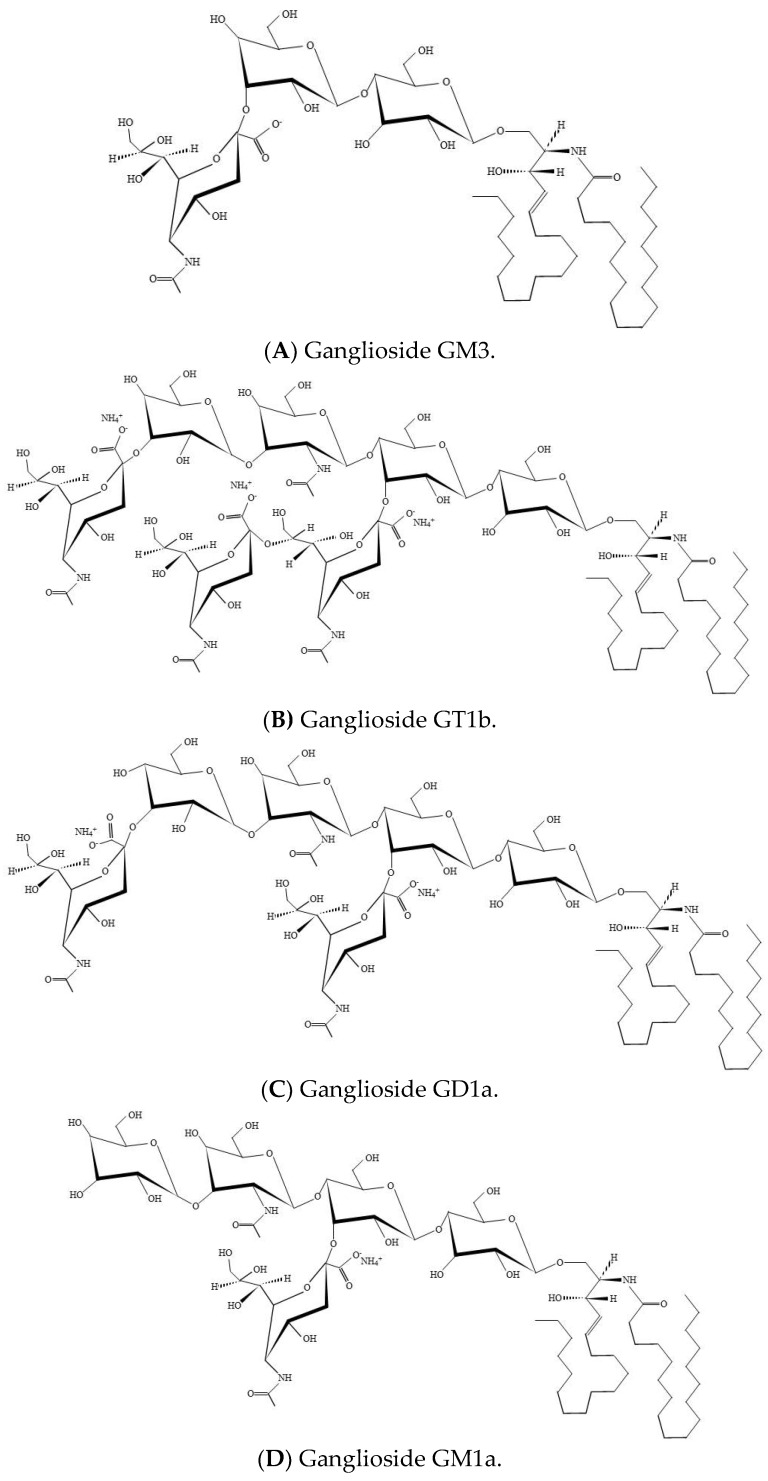
Ganglioside structure. (**A**) Structure of GM3 monoganglioside. (**B**) Structure of trisialoganglioside GT1b. (**C**) Structure of GD1a. (**D**) Structure of GM1a.

Gangliosides have been shown to modulate growth factor receptor activity, such as epidermal growth factor receptor (EGFR), platelet-derived growth factor receptor (PDGFR), and fibroblast growth factor receptor (FGFR) [24,25]. EGFR activation via EGF or EGF-like factors is recognized to impact the resumption of oocyte meiosis and cumulus cell expansion in rodents [26,27], pigs [28,29], and humans [30]. Expression of exogenous GM3 in the brain inhibits cell proliferation and induces apoptosis [31,32]. GM3 (Figure 1A) binds to the extracellular EGFR domain and inhibits the tyrosine kinase activity of EGFR. Addition of GM3 to the culture medium inhibits EGFR activity in a human epidermoid carcinoma cell line [33] and reduces the invasive potential of bladder tumors in mice [34]. GM3 also inhibits the tyrosine kinase activity of the EGFR in hepatoma cells [35] and induces cumulus cell apoptosis during in vitro maturation (IVM) of porcine oocytes. [36]. However, the growth of Siat9 (encoding GM3 synthase) and Galgt1 (encoding GM2 synthase)-deficient knock out cancer cells is strongly impeded both in vivo and vitro [37]. GM3 has also been known as an inhibitor of insulin receptor signaling. GM3 has been detected within pancreatic tissue, and pancreatic damage is related to increased GM3 abundance in streptozotocin (STZ)-induced diabetic rats [38,39]. 

Fertilization failure occurs with normal sperm and meiotically mature oocytes after conventional insemination in vitro. This unexpected outcome significantly reduces the chance of successful pregnancy. GM1 is a lipid raft and is involved in a variety of cell surface activities such as intracellular interactions, signal transduction, protein binding, and virus docking. Fertilization failure relates to defects in lipid raft microdomains enriched in GM1 at the level of the human oolemma in vitro [40]. Improved mechanisms to correlate the metabolism and molecular interactions of gangliosides with their biological functions will provide new probabilities for considering cellular regulation and will allow the manipulation of gangliosides for therapeutic advantage. This review concentrates on the effects of gangliosides in germ cells and preimplantation embryos.

## 2. Gangliosides

Glycosphingolipids (GSLs) are expressed on vertebrate cell membranes [41]. The main structure of GSLs, glucosylceramide, is synthesized on the cytoplasmic surface of the Golgi by glucosyl-ceramide synthase (GCS) via the transfer of a glucose residue from UDP-glucose to ceramide [42,43]. GCS is a transmembrane protein with its C-terminal catalytic domain located in the cytoplasm [44]. After glucosylceramide is synthesized and translocated into the Golgi lumen, it is modified by a series of Golgi glycosyltransferases to construct higher-order GSL structures [45]. Ceramide is described as an apoptotic trigger in some environments [46,47] and the apoptosis of germ cells and embryos can be caused by glucosylceramide synthase insufficiency.

Gangliosides are sialic acid-containing glycosphingolipids and are cell type-specific. Sialic acids are a large group of nine carbon α-keto acids that play a diversity of biological functions in cells [48,49]. Depending on the number of sialic acid residues on the inner galactose, gangliosides belong either to the a-ganglio series, the b-ganglio series, or the c-series [50]. GM1, related to the a-series of neuronal gangliosides, was the first complex ganglioside for which accurate chemical structure was explained [51], whereas GT1b (Figure 1B) possess three sialic acid residues; two connected to the internal galactose and one on the terminal galactose [2]. GM1 deficiencies have been detected in Huntington`s disease and Parkinson`s disease, while GM1 distribution and expression are shown to be affected in central nervous system injury caused by trauma or disease [52]. GD1a (Figure 1C) is specifically produced by the adding of sialic acid to GM1a (Figure 1D) by the synthesizing enzyme ST3 β-galactoside α-2, 3-sialyltransferase 2 (ST3GAL2) [24]. Gangliosides engage in membrane microdomains, serve as ligands of lectins, and modulate the activity of membrane proteins, such as the insulin receptor, leptin receptor, and EGFR [53,54,55,56,57,58,59,60]. In addition, gangliosides are a universal element of cell membranes with pleiotropic roles controlling intercellular activities during embryonic development, including those involved in reproductive processes [21,60]. GSL synthesis is essential for the differentiation of certain tissue and early embryonic development and supports the concept that GSLs are involved in crucial cell interactions mediating these processes [21].

## 3. Protective Effect of Gangliosides in Germ Cells and Early Embryos

**Figure 2:**
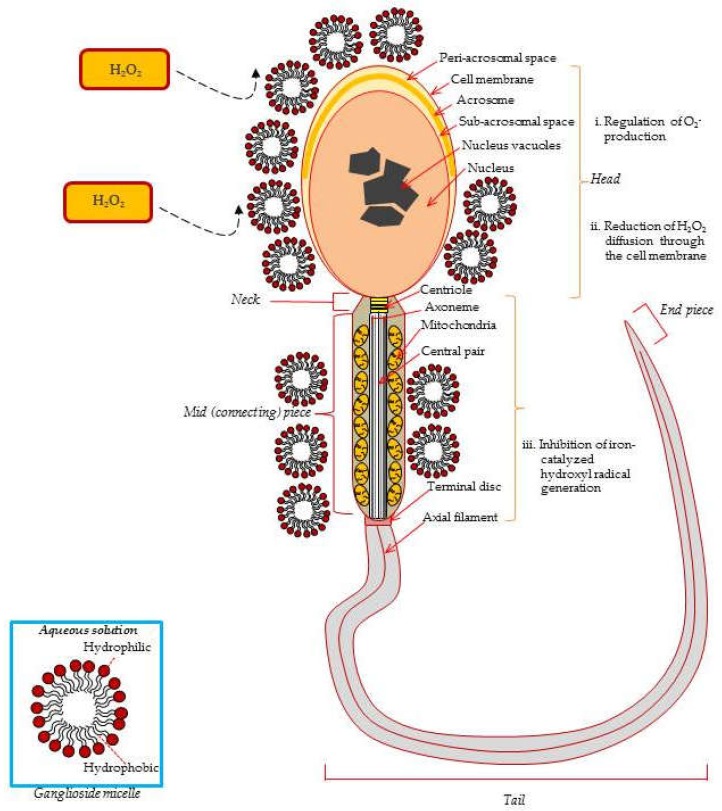
Protective effects of exogenous ganglioside micelles from ROS-induced damage in the ejaculated spermatozoa. Exogenous gangliosides are absorbed to the sperm surface and reduce the superoxide anion level generated by PMA-stimulated spermatozoa. O^-^_2_ is converted into H_2_O_2_ by cytosolic-localized SOD1 (Cu, Zn, SOD), mitochondria-localized SOD2 (Mn SOD), and the extracellular SOD3 (Fe SOD). Ganglioside GT1b reduces Fe^2+^-mediated decomposition of lipid hydroperoxydes from the sperm membrane. Consequently, GT1b prevents lipid peroxidation induced sperm membrane damage due to its specific molecular structure, such as micelles [16,20,70].

Excessive ROS generation can increase DNA injury in the cell, including sperm, oocytes, and embryos. Mono-, di-, and trisialogangliosides have been shown to decrease the lipid peroxidation process (LPO) induced in brain synaptosomes by the ferrous-ascorbate induced lipid peroxidation [18] and to scavenge free radicals generated during myocardial ischemic reperfusion [61]. Yamamoto et al. [21] demonstrated that ganglioside GT1b suppressed brain mitochondrial DNA damage. Exogenously added GD1b and GT1b reduced the level of superoxide anions produced by phorbol,12-myristate acetate (PMA)-stimulated human sperm [18]. Gavella et al. [62] reported that ganglioside micelles attached to the ejaculated sperm membrane to provide a diffusion barrier and delayed ROS production (Figure 2). Moreover, exogenous GT1b increases the oocyte nuclear maturation rate by reducing ROS during porcine IVM [62].

Cryopreservation can be valuable for assisted reproductive technologies (ART) to preserve fertility; however, cryopreservation significantly increases DNA damage in cells. Several reports have proposed that DNA fragmentation can be mainly assigned to cryopreservation-produced ROS [63,64,65]. We reported that ganglioside GT1b was expressed in surviving murine embryos during the development of both fresh and frozen embryos [66]. These results demonstrate that GT1b suppresses DNA damage and participates in embryo survival and growth. During cryopreservation, a major site of injury is the plasma membrane. Gangliosides are involved in multiple functions, and it is important to understand how their distribution is modulated in the plasma membrane.

Cryopreservation-produced injury to the spermatozoa membrane manifests as changes in the organization and lipid arrangement of the membrane, leading to changes in spermatozoa permeability [67]. One of the suggested processes of spermatozoa defiance to cryostorage-produced damage is its ability to shed hydrophilic lipids, therefore increasing the hydrophobic features of the membrane and increasing spermatozoa tolerance to cryopreservation [68]. Gavella et al. [23] showed that treatment of GM1 and GT1b defended human spermatozoa DNA integrity during the freezing–thawing procedures. GM1-pretreated cryopreserved embryonic ventral mesencephalon (VM) plus a daily treatment with GM1 to the culture medium improves the low survival and functional inefficacy of grafts derived from cryopreserved VM in rats [69].

## 4. Effects of Gangliosides on Spermatozoa.

Spermatozoa undergo three distinct phases of maturation; spermatogenesis, epididymal maturation, and capacitation. Spermatogenesis occurs in the male testis and is responsible for the production of spermatozoa. Spermatozoa are able to swim and fertilize an oocyte after maturing in the epididymis. The epididymis is a duct and is subdivided into the head region, corpus region, and the tail region. Seminiferous tubules are located in the testis and new spermatogonia begin their division in the seminiferous tubules. In rat seminiferous tubules, GM3 is expressed in pachytene spermatocytes and spermatids; however, GM3 is not detected in spermatogonia [71]. It is suggested that GM3 might play a key role in maturation of spermatozoa and obtain a fertility ability. β1,4-*N-*Acetylgalactosaminyltransferase (*β1,4GalNAc-T*) is an important enzyme in the biosynthesis of complex gangliosides, because all complex gangliosides are synthesized through GM2 or GD2. Mice with a disrupted *β1,4GalNAc-T* gene allele, and the knockout mouse line of the *β1,4GalNAc-T* gene lack all complex ganglio-series gangliosides [72]. Given the fact that several seminiferous tubules showed entire lack of the germ cell in the *β1,4GalNAc-T* gene KO male mice, the result suggested that gangliosides are essential in the transport of testosterone to the seminiferous tubules and bloodstream from the Leydig cells in vivo [73]. 

Sperm capacitation is the process that confers the acquisition of fertilization-competence in the female reproductive tract and correlated with changes in sperm intracellular ion concentrations, metabolism, the plasma membrane, and motility [74,75,76]. Cholesterol is the major sterol in animal spermatozoa and intercalates between glycolipids and phospholipids in the hydrophobic interior of the cell membrane and increases or decreases order, depending on the degree of saturation or unsaturation of the fatty acyl chains [77]. GM1 localizes to the sterol-rich plasma membrane overlying the acrosome of sperm. During capacitation, GM1 localizes to the sterol-poor postacrosomal plasma membrane and then moves to the sterol-rich plasma membrane in rat spermatozoa [78]. The distribution of GM1 on ejaculated boar spermatozoa changes in a sequential manner from overlying the sperm tail to the sperm head during methyl-β-cyclodextrin(MBCD)-mediated capacitation [79]. In live bovine and mouse sperm heads, GM1 also localizes to the sterol-rich plasma membrane overlying the acrosome (APM), and labeling GM1 using the pentameric cholera toxin subunit B (CTB) induces a dramatic redistribution of signal from the APM to the sterol-poor postacrosomal plasma membrane upon sperm death [80]. In this context, the Cap-Score^TM^ Sperm Function Test (Cap-Score) is a laboratory-developed test that is designed to assess sperm capacitation in vitro [81]. This assay therefore analyzes the GM1 localization patterns to evaluate the fertilizing ability of human sperm. Changes of GM1 may play an important role in mediating sperm–oocyte interactions in mammalian spermatozoa.

## 5. Gangliosides on Oocytes and Preimplantation Embryos

Sex hormones such as follicle stimulation hormone (FSH) and luteinizing hormone (LH) stimulate the continuation of the first meiotic division of oocytes and oocyte maturation, including nuclear and cytoplasmic changes during the developing gametes. During the oestrous cycle, follicular development, ovulation, and luteinization are regulated by cell–cell interactions and hormones, implying the importance of surface membrane components including gangliosides. GM1 and GM3 are expressed in theca cells during rat follicular development [82]. Cumulus cells provide metabolic and nutritional support for the oocytes. Cumulus expansion is essential for ovulation and influences oocyte maturation. In the hypophysectomized rat ovary, it has been shown that GM3 is expressed in cumulus and granulosa cells of Graafian follicles after gonadotropin stimulation, and these expression patterns are quite similar to those of normal adult rats [83].

**Figure 3:**
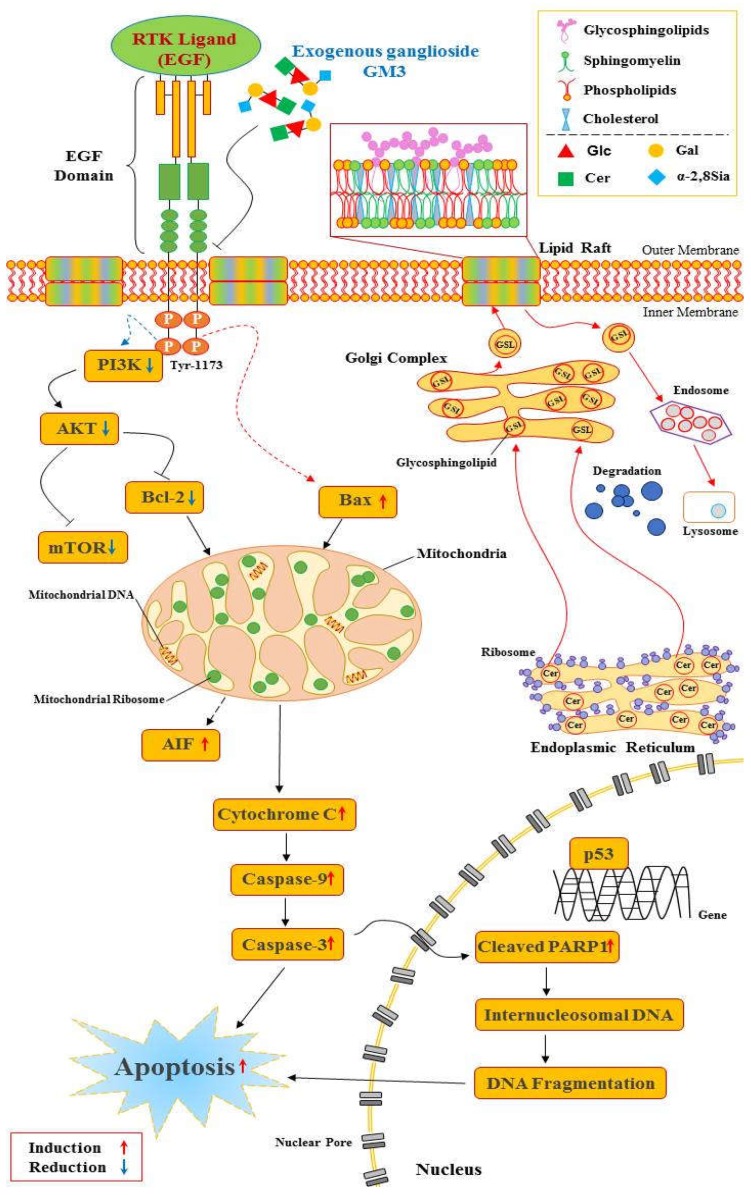
Exogenous GM3 and the EGFR-mediated PI3K/AKT signal pathway. Exogenously added GM3 binds to the extracellular domain of EGFR and inhibits its dimerization [104] without inhibiting ligand binding [105]. Exogenous GM3 inhibits EGF-induced phosphorylation of EGFR at the Tyr-1173 residue [35]. After inhibition by blocking phosphorylation, the inhibited signal from the EGFR prevents one of the three downstream signaling pathways, PI3K, AKT, or mTOR. PI3K and AKT activation is reduced [106]. Upon inhibition of the phosphorylation of PI3K, AKT subsequently suppresses the phosphorylation of mTOR, which mediates protein synthesis [107]. This mechanism for suppressed EGFR activity might reduce cell proliferation and inhibit the repair of DNA damage [108]. The activation changes of these pro- and anti-apoptotic proteins is initiated in mitochondria [109]. Continuous mitochondrial regulation releases pro-apoptotic proteins such as cytochrome c into the cytoplasm causing caspase cascades processed by apoptosis, the programed cell death [52].The most compelling evidence shows that GM3 treatment decreases phosphorylated EGFR levels and regulates down-stream EGFR activation in cumulus-oocyte complexes COCs. Consequently, GM3 exposure reduces EGFR-delivered PI3K/AKT signaling for proliferation during in vitro maturation of porcine COCs [36].

EGFR is one of the receptor tyrosine kinases, and activation of EGFR induces numerous signaling pathways related to cell proliferation and resistance to apoptosis [22]. In this context, the exogenous GM3 inhibits EGF-induced phosphorylation of the EGF receptor at the Try-1173 residue, but enhances the phosphorylation of the Try-1088 residue [35]. Under these circumstances, both Try-1173 and Try-1086 residues serve as a docking site for the SH2-domain containing signaling molecules and lead to activation of the downstream signaling pathways. Similarly, GM3 inhibits c-fos and c-jun expression, and thus consequently the inhibition of c-fos and c-jun by GM3 is consistent with the inhibition of MAP kinase activity by GM3 [84]. Additionally, Park et al. [36] showed that in porcine cumulus-oocyte complexes (COCs), exogenous GM3 inhibits meiotic maturation and restricts the expansion of cumulus cells during in vitro meiotic maturation through EGFR-mediated PI3K/AKT signaling pathways and initiates apoptosis (Figure 3). GM3 also works and shows increased apoptosis in human colon cancer [85] and murine bladder cancer [34]. Addition of GM3 inhibits insulin-stimulated phosphorylation of the insulin receptor in 3T3-L1 and autophosphorylation of soluble receptors. In other words, the GM3 synthase knockout mice enhance ligand-induced insulin receptor phosphorylation and sensitivity to glucose [86]. Ju et al. [87] demonstrated that ganglioside GM3 expression increased in apoptotic preimplantation embryos. In relation to this, GM3 is involved in transmembrane signaling modulation of growth factor receptor activities, and in cell adhesion and motility, whereby GM3 expression is decreased during ovarian maturation and early embryonic development in the diabetes mouse [88].

Lipid rafts play a role in a number of signaling processes involving receptors that are expressed by a variety of cell types, including the EGFR receptor, integrin, and the insulin receptor. Ganglioside GM1 dramatically inhibits EGFR activation by changing the distribution of EGFR from the glycospingolipid-enriched microdomain (GEM) domain in human mammary epithelial cells [89]. These particular microdomains allow plasma membrane subcompartmentalization and the establishment of signaling platforms that intermediate physiological reactions. Mirkin et al. [90] noted that GM1 has a weak capacity to inhibit human neuroblastoma cell proliferation and EGFR phosphorylation. Moreover, treatment with GM1 significantly reduces phosphorylation of PDGFR by excluding the PDGFR from GEM domains [91]. For this reason, the overexpression of GM1 suppresses TrkA activiation by modulating the distribution of receptors from the lipid raft fraction to the non-raft fraction in PC12 cells [92]. Through this lens, it is seen that lipid rafts play an important role in cleavage furrow ingression and central spindle assembly. Toward this reasoning, GM1 is considered one of the important marker GSLs for lipid rafts, and it is easily detected with cholera toxin B subunit (CTX-B). Additionally, it is shown that GM1 detected with biotinylated CTX-B is enriched at the cleavage furrow in mouse 2-cell and 4-cell embryos that undergo cytokinesis [93]. GM1 accumulates in the perivitelline space in unfertilized mouse oocytes, and its accumulation increases after fertilization [94]. In consideration of this, GM1 microdomains are involved in the early stage of the fertilization procedures by supplying docking sites for spermatozoa in the human oolemma [40]. Bouvier et al. [95] demonstrated that predominant gangliosides were GD3 (52%), GM3 (19%), and GT1b in the E-12 mouse embryo, while b-series gangliosides, such as GD3, GD1b, GT1b, and GQ1b significantly reduced in the twl/twl mutant mouse. Ganglioside GM3 and GD3 are markedly expressed in mid-embryonic brains, but their expression is significantly decreased during later development when a- and b-series gangliosides are increased [96].

Calmodulin (CaM) is the major intracellular receptor for Ca^2+^ and regulates various intracellular enzymes that include protein phosphatases, ion channels, and protein kinases [97]. The CaMKII family of serine/threonine protein kinases regulates the transition of metaphase into anaphase in the cell cycle and has 28 isoforms originating from α, β, γ, and δ genes [98]. Here it is shown that CaMKII is involved in activation-like regulation of other proteins and meiotic resumption in porcine oocytes [99]. Moreover, in mouse oocytes, CaMKII also interacts with molecules that control cell cycle and Ca^2+^ signaling in mouse oocytes [100]. To be sure, CaMKII γ and CaMKII δ are expressed in porcine cumulus cells and oocytes [62]. Incidentally, it is noted that intracellular Ca^2+^ levels are increased with a higher concentration of GT1b in a dose-dependent manner during oocyte IVM [62]. The results show that the addition of GT1b to the culture medium temporarily activates CaMKII in neuroblastoma-glioma hybridoma (NG108-15) cells or rat hippocampal cells [101]. Several gangliosides, including GD1a play an important role in oocyte maturation via regulation of intracellular Ca^2+^ or EGF-induced EGFR phosphorylation. Exogenously added GD1a into culture medium promotes oocyte maturation and preimplantation development ability via stimulation of oocyte meiosis and maturation. On the other hand, exogenous GD1a decreases polyspermic fertilization in pigs [102]. Choo et al. [82] showed that GD1a is distributed within theca cells, interstitial cells, and oocytes during ovarian maturation in the rat. In addition, pre-treatment of GD1a increases EGFR dimerization, EGF-induced EGFR autophosphorylation, and receptor-tyrosine kinase activity in normal human dermal fibroblasts [103].

## 6. Conclusions

Gangliosides, which are sialic acid-containing glycosphingolipids, can be antioxidant candidates whose in vivo and in vitro applications have been demonstrated to efficiently protect sperm, oocytes, and embryos from ROS-produced injury. The preserving role of ganglioside GT1b against ROS-produced shifts is considered to originate from its ability to scavenge free radicals and protect from injury, whereas ganglioside GM3 induces apoptotic cell death in early embryos. To put it differently, exogenously added gangliosides improve oocyte maturity, monospermic fertilization, and preimplantation embryonic development.

Furthermore, recent work has placed gangliosides, with their various glycan structures spreading into the extracellular space and their lateral relations with signaling molecules in the cell membrane, among the significant controlling components involved in cell signaling and cell–cell recognition. Nevertheless, the important roles of gangliosides in the cell membrane require further investigation. Largely, it has been noted that ganglioside activity may contribute to the development of optimal cryoprotection techniques to preserve DNA integrity and culture conditions for assisted reproduction therapy.
